# 

**Published:** 1917-12

**Authors:** 


### CONTENTS.

*Page*

War Surgery in an Indian General Hospital in Mesopotamia. By PERCIVAL S. CONNELLAN, I.M.S. ... ... ... ... ... 113

Reviews of Books ... ... ... ... ... ... ... ... 129

Obituary Notices:

W. J. HILL, M.R.C.S., L.R.C.P. ... ... ... ... ... ... 136

W. J. H. PINNIGER, M.D. ... ... ... ... ... ... ... 136

